# Machine Learning Prediction Models for Mobility Limitation Over Time in Older Adults: The Health ABC Study

**DOI:** 10.1093/geroni/igab046.094

**Published:** 2021-12-17

**Authors:** Jaime Speiser, Kathryn Callahan, Edward Ip, Michael Miller, Janet Tooze, Stephen Krtichevsky, Denise Houston

**Affiliations:** 1 Wake Forest School of Medicine, Winston-Salem, North Carolina, United States; 2 Wake Forest School of Medicine, Winston Salem, North Carolina, United States; 3 Wake Forest School of Medicine, Wake Forest School of Medicine, North Carolina, United States

## Abstract

Mobility limitation in older adults is common and associated with poor health outcomes and loss of independence. Identification of at-risk individuals remains challenging because of time-consuming clinical assessments and limitations of statistical models for dynamic outcomes over time. Therefore, we aimed to develop machine learning models for predicting mobility limitation in older adults using repeated measures and variable selection. We used nine years of follow-up data from the Health, Aging, and Body Composition study to model mobility limitation, defined as self-report of any difficulty walking ¼ mile or up a flight of stairs, assessed annually. We considered 46 predictors for modeling, including demographic, lifestyle, chronic condition and physical function variables. We developed three models with Binary Mixed Model Forest, using: 1) all 46 predictors, 2) an automated variable selection algorithm, and 3) the top five most important predictors. Area under the receiver operating curve ranged from 0.78 to 0.84 for the models for two validation datasets (with and without previous annual visit data for participants). Across the three models, the most important predictors of mobility limitation were ease of getting up from chair, gait speed, self-reported health status, body mass index and depression. Longitudinal, machine learning models predicting mobility limitation had good performance for identifying at-risk older adults based on current and previous annual visit data. Future studies should evaluate the utility and efficiency of the prediction models as a tool in a clinical setting for identifying at-risk older adults who may benefit from interventions aimed to prevent mobility limitation.

